# Identifying primary care patients at risk for future diabetes and cardiovascular disease using electronic health records

**DOI:** 10.1186/1472-6963-9-170

**Published:** 2009-09-22

**Authors:** Marie-France Hivert, Richard W Grant, Peter Shrader, James B Meigs

**Affiliations:** 1 General Medicine Division, Massachusetts General Hospital, and Harvard Medical School, Boston, MA, USA

## Abstract

**Background:**

Prevention of diabetes and coronary heart disease (CHD) is possible but identification of at-risk patients for targeting interventions is a challenge in primary care.

**Methods:**

We analyzed electronic health record (EHR) data for 122,715 patients from 12 primary care practices. We defined patients with risk factor clustering using metabolic syndrome (MetS) characteristics defined by NCEP-ATPIII criteria; if missing, we used surrogate characteristics, and validated this approach by directly measuring risk factors in a subset of 154 patients. For subjects with at least 3 of 5 MetS criteria measured at baseline (2003-2004), we defined 3 categories: *No MetS *(0 criteria); *At-risk-for MetS *(1-2 criteria); and *MetS *(≥ 3 criteria). We examined new diabetes and CHD incidence, and resource utilization over the subsequent 3-year period (2005-2007) using age-sex-adjusted regression models to compare outcomes by MetS category.

**Results:**

After excluding patients with diabetes/CHD at baseline, 78,293 patients were eligible for analysis. EHR-defined MetS had 73% sensitivity and 91% specificity for directly measured MetS. Diabetes incidence was 1.4% in *No MetS*; 4.0% in *At-risk-for MetS; *and 11.0% in *MetS *(p < 0.0001 for trend; adjusted OR *MetS *vs *No MetS *= 6.86 [6.06-7.76]); CHD incidence was 3.2%, 5.3%, and 6.4% respectively (p < 0.0001 for trend; adjusted OR = 1.42 [1.25-1.62]). Costs and resource utilization increased across categories (p < 0.0001 for trends). Results were similar analyzing individuals with all five criteria not missing, or defining MetS as ≥ 2 criteria present.

**Conclusion:**

Risk factor clustering in EHR data identifies primary care patients at increased risk for new diabetes, CHD and higher resource utilization.

## Background

Identifying individuals at risk for chronic diseases is the first step toward preventive measures. Metabolic syndrome is a diagnosis that has been proposed to identify patients in whom the clustering of risk factors is associated with increased risk of diabetes and cardiovascular disease [1]. The risk factors included in the National Cholesterol Education Program-Adult Treatment Panel III (NCEP-ATPIII) definition are central obesity (measured by waist circumference), dyslipidemia (high triglycerides and low HDL), impaired glucose metabolism, and elevated blood pressure. Although the underlying cause for their clustering is not understood, these risk factors cluster together more often than predicted by chance alone [2]. National surveys and large population-based studies have shown that metabolic syndrome is common [3,4] and is associated with substantial health care costs [5]. Data from the NHANES III estimated that 24% of the US population over the age of 20 fulfilled the criteria of the metabolic syndrome according to the NCEP-ATPIII definition [3]. The clinical usefulness of the metabolic syndrome diagnosis has been debated [6,7] but in fact, very few studies have reported data on metabolic syndrome in real clinical practice settings. The concept of risk factor clustering has high potential for identification of at-risk patients, but data from real-world clinical care is needed to understand the actual usefulness of the metabolic syndrome concept as a marker of risk factor clustering and a target for prevention of its adverse consequences.

Many hospitals and outpatient settings now have electronic-based health records (EHR) that can be queried for clinical care research and quality improvement [8]. The clinical information recorded in the EHR could be used for identification of populations at risk who might benefit from targeted preventive interventions. The opportunity to use the EHR to identify risk factor clustering and metabolic syndrome has not been investigated. From a public health perspective, it would be efficient to use already-collected clinical care information to identify individuals at risk for developing chronic disease.

With this in mind, we hypothesized that: 1) we could identify people with metabolic syndrome in the EHR of our large primary care practice-based network, even when considering the limitation of missing or misclassified data; 2) metabolic syndrome would be associated with higher health care utilization and costs than for people without metabolic syndrome; and 3) people with metabolic syndrome would be at increased risk for subsequent development of diabetes or coronary heart disease (CHD) relative to people without metabolic syndrome. Our aim was to assess whether risk factor clustering identified in EHR data identifies increased-risk people who might subsequently benefit from prevention interventions.

## Methods

### Data Source and Study Patients

We identified all people receiving regular care from an identified primary care physician in a network of 12 outpatient practices in eastern Massachusetts affiliated with Massachusetts General Hospital (MGH) and the Partners Healthcare System (PHS): the MGH Primary Care Practice-Based Research Network (PBRN). PBRN practices include three hospital-affiliated academic practices, four community health centers, and five private practice offices, together serving a wide range of communities and patient populations. PBRN practices share a common EHR containing all clinical and utilization data for each patient. EHR data are searchable in the Research Patient Data Repository (RPDR) . Using the RPDR we selected for study those patients older than 18 years (age range from 18 to 105 years old), with at least one outpatient visit between January 1^st^, 2003 and December 31^st^, 2004. We queried coded field data for physical examination, medication lists, problem lists, clinical laboratories, demographic information including race, insurance status, home zip code (to calculate median household income based on Federal tax return data for that code), and health care utilization and cost information. The study was approved by the Massachusetts General Hospital/Partners Health Care System Institutional Review Board.

### Diagnosis of Diabetes and Coronary Heart Disease

We excluded individuals with diabetes and/or coronary heart disease (CHD) at baseline from the primary analysis because we wanted to assess incident diagnosis of these conditions, and because our approach is intended to be used for prevention of these conditions. Baseline diabetes diagnosis was defined using a previously validated algorithm that included diabetes mellitus on the problem list, diabetes-specific medications, hemoglobin A1c (HbA1c) results > 7.0%, or one inpatient diagnosis code or two outpatient diagnosis codes for diabetes (ICD-9 codes 250.xx). This algorithm has 98% sensitivity and 98% specificity for diabetes when compared to the gold standard of manual chart review by a trained research nurse [9]. We did not further discriminate type 1 from type 2 diabetes. Baseline CHD diagnosis was based on presence of coronary heart disease defined by any one of the following three criteria: 1) one inpatient diagnosis code or two outpatient diagnosis codes for either coronary artery disease or myocardial infarction (ICD-9 codes 410.x through 414.x and 429.2); 2) Current Procedural Terminology (CPT) billing codes for coronary artery bypass grafting (CABG) or percutaneous insertion of an intracoronary stent; and/or 3) Evidence of myocardial infarction by elevated troponin T (greater than 0.09 ng/ml on one or more occasions). The approach has a sensitivity of 100% and specificity of 97% for CHD when compared to the gold standard of detailed manual chart review [10].

### Definition of Formal and Surrogate Characteristics

The thresholds for criteria used in each of the five categorical characteristics of the metabolic syndrome are presented in Table 1. We used the updated NCEP-ATPIII thresholds to define formal criteria. If formal criteria were not available in the EHR, we queried for surrogate criteria. Central obesity surrogate criteria cut-offs were based on reported BMI equivalents for waist circumference in men and women of the Framingham Offspring Study [11]. The BMI cut-offs for men (29.1 kg/m^2^) and for women (27.2 kg/m^2^) corresponded to the 66^th ^percentile in the PBRN population. If height was not available in the dataset, we used the 66^th ^percentile of weight in both sexes (≥ 201 lbs in men, ≥ 162 lbs in women). The surrogate criteria for non-fasting glucose, triglycerides and total cholesterol were defined by thresholds used in the original, non-fasting Framingham Heart Study cohort analyses [12]. If more than one measurement for a specific criterion was available during the baseline period, the most recent measurement was used.

**Table 1:** Formal and surrogate metabolic syndrome characteristics used to define electronic health record metabolic syndrome categories.

**Metabolic syndrome criteria**	**Formal NCEP-ATPIII characteristic**	**Surrogate characteristic**
Central obesity	Waist circumference >102 cm (40") in men, >88 cm (35") in women	Body mass index ≥ 29.1 kg/m^2 ^in men, ≥ 27.2 kg/m^2 ^in women; if height missing, weight ≥ 94.4 kg (201 lbs) in men, ≥ 73.6 kg (162 lbs) in women*

Elevated blood pressure ‡	Average of last two readings ≥ 130 and/or ≥ 85 mmHg or diagnosis of hypertension on problem list and antihypertensive agent on medication list	Any of: one blood pressure ≥ 130 and/or ≥ 85 mmHg; on anti-hypertensive medication but no diagnosis on problem list; hypertension from billing codes

Elevated glucose	Fasting plasma glucose >5.6 mmol/L (100 mg/dL)	Any plasma glucose ≥ 7.8 mmol/L (140 mg/dL)

Elevated triglycerides	Fasting plasma level ≥ 1.7 mmol/L (150 mg/dL)	Any plasma level ≥ 2.3 mmol/L (200 mg/dL)

Low HDL cholesterol	Plasma level <1.0 mmol/L (40 mg/dL) in men, <1.3 mmol/L (50 mg/dL) in women	Plasma total cholesterol >5.2 mmol/L (200 mg/dL)

*BMI cut-offs chosen based on report of waist circumference equivalent from the Framingham Offspring Study; weight cut-offs corresponding to the 66th percentile of available weights in the population (percentile based on the percentile of the values for the cut-offs for BMI)

‡ All patients in our dataset had available data to assess formal criteria of blood pressure, so the surrogate criteria were not used in the present analysis

### Classification of Metabolic Syndrome

After excluding patients meeting our EHR diagnostic criteria for diabetes or CHD, we assessed the presence of measured metabolic syndrome criteria in the remaining patient population. Once all the patients were assessed for presence or absence of measurement in the five categories of criteria, we restricted our analysis to those patients with at least three criteria measured. We then categorized patients into three groups: 1) No metabolic syndrome (zero risk factors present); 2) At-risk-for metabolic syndrome (1 or 2 risk factors present); and 3) having Metabolic syndrome (≥ 3 risk factors present).

### Validation of criteria and Metabolic Syndrome classification

We recruited 154 patients in one PBRN practice, the MGH Internal Medicine Associates (IMA), to validate our approach to classifying EHR metabolic syndrome criteria. We invited patients to arrive 30 minutes before the time of their regular scheduled appointment to have standardized assessment of risk factors. Height and weight were measured without shoes in light street clothing; waist circumference was measured above the iliac crest and the average of two measurements was used. Blood pressure was measured after the patient had been sitting for at least 5 minutes; the average of the two measurements, taken at least 5 minutes apart, was used. Blood glucose and lipids were drawn after an overnight fast of at least eight hours.

The thresholds for BMI and weight in the EHR used as surrogate criteria (see Table 1) had a sensitivity of 88% and specificity of 87% (c-statistic = 0.876) to predict central obesity defined by waist circumference >40" in men, and >35" in women. The other criteria had c-statistics between 0.678 and 0.855 (see Additional file 1: Table S1 for details on each criterion). Meeting at least 3 criteria in the EHR had a sensitivity of 73% and a specificity of 91% for detecting metabolic syndrome measured in a standardized fashion (c-statistic = 0.818). These validation data confirmed that we could use the EHR to identify reliably individuals with metabolic syndrome in primary care practices.

### Outcomes assessment

We queried the RPDR database over the time period from January 1^st ^2005 up to December 31^st^, 2007. The RPDR includes patient utilization, cost, problem and discharge diagnosis lists, and hospital-based medication data. We used the RPDR to query billing data (principal and secondary diagnoses and procedures, inpatient and outpatient total, direct, and itemized costs, hospital charges including provider, type of service and date) and other administrative information (primary care physician, hospital admissions and emergency room visits, hospital length of stay, admission service, and discharge disposition). The health resources-related outcomes of interest included the number of hospital inpatient admissions, total cost per admission, length of stay, and number of outpatient visits. New diagnoses of diabetes and CHD over the 3-year follow-up were identified using the same validated algorithms described above.

### Statistical analysis

We restricted our analysis to those patients with at least three measured metabolic syndrome traits, in the framework of case finding in a broad population of usual care patients where some missing data are expected. Primary analyses were conducted using metabolic syndrome defined as having three or more risk factors present. Outcomes were log-transformed to improve normality when appropriate. Health resource outcomes were analyzed using age-sex adjusted linear regression models; p-values are reported for trend across metabolic syndrome categories (*No metabolic syndrome*, *At-risk-for metabolic syndrome*, and *Metabolic syndrome*) or across four categories (the three metabolic syndrome categories plus patients with diabetes and/or CHD at baseline). Diabetes and CHD incidence were analyzed using age-sex-adjusted logistic regression models; p-values are reported for trends across the three metabolic syndrome categories. Odds ratios (age-sex-adjusted) were calculated to compare patients in the *Metabolic syndrome *or the *At-risk-for metabolic syndrome *groups to individuals in the *No Metabolic syndrome *group. Population attributable risk proportion (PAR) was calculated using the formula: pd ([RR-1]/RR) where pd = proportion of cases exposed to the risk factor, and RR = relative risk [13].

We conducted subsidiary analyses using only the patients having all five criteria measured to assess prevalence of metabolic syndrome and to compare our population to national data. Another set of subsidiary analyses was conducted to assess outcomes using a more sensitive (but less specific) approach, to consider the perspective of identifying a wider population that might benefit from larger scale prevention approaches. For this, we used the population with at least 3 criteria measured but defined the presence of metabolic syndrome as meeting two or more criteria. With this approach, EHR metabolic syndrome (≥ 2 criteria) had a sensitivity of 91% and a specificity of 76% for directly-phenotyped metabolic syndrome (≥ 3 criteria). Finally, we conducted a sensitivity analysis using all patients in the dataset (including individuals with less than 3 criteria measured).

We used SAS for all analyses (SAS v 9.1, SAS Institute Inc., Cary, North Carolina), and considered p-values < 0.05 to indicate statistical significance. The authors had full access to the data and take responsibility for its integrity.

## Results

There were 122,715 patients available for analysis in the baseline period, including 17,320 patients with diabetes and/or CHD. The remaining 105,395 patients were evaluated for the presence of measured formal or surrogate metabolic syndrome criteria as defined in Table 1. In this population, 0% had a waist circumference recorded; triglycerides and glucose were measured in the fasting state in only 21.0% and 17.2%, respectively. Distributions of the formal and surrogate criteria among all patients without diabetes or CHD and among those with at least three criteria measured are shown in Additional file 1: Table S2. Of the 105,395 patients without diabetes or CHD, 78,293 patients had at least 3 metabolic syndrome criteria measured and are the subject of this analysis. Demographic characteristics are presented in Table 2. Individuals in the *Metabolic syndrome *category were older, more likely to be male, more likely to be insured by Medicare, and to have a lower mean annual income compared to patients in the *No metabolic syndrome *or *At-risk for metabolic syndrome *categories.

**Table 2:** Baseline characteristics and three-year health care utilization (2005-2007) for individuals with *No metabolic syndrome*, *At-risk-for metabolic syndrome*, with *Metabolic syndrome*, and with diabetes and/or coronary heart diseases (CHD) according to electronic health record data

	No metabolic syndrome*	At-risk-for metabolic syndrome*	Metabolic syndrome*	**Any diabetes/** **CHD**		
	**0 criteria**	**1-2 criteria**	**≥ 3 criteria**			

***Baseline characteristics***						

N	36,841	36,267	5,185	17,320		
Age, years (SD)	45 (15)	49 (16)	52 (14)	64 (16)		
Women, %	62.8%	57.4%	48.6%	48.0%		
Race (% White)	78.8%	76.5%	78.5%	79.3%		
Insurance						
Insurance % Commercial	77.4%	66.1%	62.2%	35.1%		
% Medicare	10.6%	18.3%	20.4%	5.4%		
Income, $US (SD)	$81,402 ($59,796)	$70,551 ($53,479)	$62,101 ($45,643)	$64,190 (49,551)		

***Three-year outcomes***					p-value†	p-value‡
Inpatient admissions, mean number per patient (SD)	0.12 (0.61)	0.23 (1.07)	0.35 (1.20)	1.5 (3.6)	<.0001	<.0001
Length of stay, mean days (SD) §	4 (3)	5 (3)	6 (3)	12 (3)	<.0001	<.0001
Outpatient clinic visits, mean number per patient (SD) §	4.3 (2.3)	6.6 (2.5)	10.4 (2.5)	20.5 (2.5)	<.0001	<.0001
Total cost, US dollars (SD) §	$3,054 ($4)	$4,136 ($4)	$5,057 ($4)	$9,177 ($4)	<.0001	<.0001

Incidence of diabetes mellitus	1.4%	4.0%	11.0%	N/A	<.0001	N/A

Incidence of CHD	3.1%	5.3%	6.4%	N/A	<.0001	N/A

* Individuals categorized after exclusion of diabetes and CHD, including only patients with at least three criteria (formal or surrogate) measured (n = 78,293) using electronic health record data from 2003-2004

† p-values for trend in age-sex-adjusted regression analysis in all 3 categories of metabolic syndrome status (*No metabolic syndrome*, *At-Risk-for metabolic syndrome*, and with *Metabolic syndrome*)

‡ p-values for trend in age-sex-adjusted regression analysis in all 4 categories (*No metabolic syndrome*, *At-Risk-for metabolic syndrome*, with *Metabolic syndrome*, and any diabetes and/or CHD)

§log-transformed for analysis and then back-transformed for display

Health care utilization from 2005 to 2007 is presented in Table 2 by metabolic syndrome category and presence of diabetes and/or CHD. Patients with diabetes/CHD used more health care resources and were more costly overall (all p-values < 0.0001). Across categories of metabolic syndrome, trends for increased number of admissions, cost per admission, length of stay, and number of outpatients visit all were significant (all p-values < 0.0001), with the patients having *Metabolic syndrome *using more health care resources than those without.

Diabetes incidence was increased across metabolic syndrome categories (p < 0.0001 for trend), with the patients in the *Metabolic syndrome *group being at the highest risk (11.0%) of new diabetes over 3 years. Compared to the *No metabolic syndrome *group, the age-sex-adjusted OR for new cases of diabetes in patients was 2.39 (2.26-2.64) for people *At-risk-for *and 6.86 (6.06-7.76) for individuals with *Metabolic syndrome *(P < 0.0001); the PAR for diabetes was 38.1% and 25.3% in each of those two categories. CHD incidence was also increased among people *At-risk-for *or with *Metabolic syndrome*, with those with *Metabolic syndrome *having the highest incidence of new CVD (6.4% over 3-year follow-up; p < 0.0001 for trend). Compared to the *No metabolic syndrome *group, the age-sex-adjusted OR for new cases of CHD was 1.20 (1.11-1.30) for people *At-risk-for *and 1.42 (1.25-1.62) for individuals with *Metabolic syndrome *(P < 0.0001); the PAR for CHD was 7.6% and 2.5% in each of those two categories.

### Subsidiary analyses

To assess whether missing data for metabolic syndrome criteria distorted the analysis in any way, we conducted subsidiary analyses among 30,461 patients who had all five criteria measured (that is, no missing data) in the EHR. Additional file 1: Table S2 shows the prevalence of patients meeting formal and surrogate criteria (after exclusion of diabetes and CHD) in the population with all five criteria measured. It is clear that missing data resulted in lower prevalences of metabolic syndrome characteristics in the classification that used patients with only three or more criteria measured. To assess effects of missing data, we repeated the main analysis using subjects with no missing data. We found that baseline characteristics and outcomes were very similar to those when found when analyzing the population of patients with at least three criteria measured (see Table 3). In this sub-group with all five criteria measured, compared to the *No metabolic syndrome *group, the age-sex-adjusted OR for new cases of diabetes in patients was 2.77 (2.25-3.42) for people *At-risk-for *and 8.67 (6.98-10.77) for individuals with *Metabolic syndrome *(P < 0.0001). Compared to the *No metabolic syndrome *group, the age-sex-adjusted OR for new cases of CHD was 1.44 (1.22-1.69) for people *At-risk-for *and 1.91 (1.58-2.32) for individuals with *Metabolic syndrome *(P < 0.0001). Thus, while missing data affected the prevalence of metabolic syndrome characteristics it had a negligible effect on the association of EHR metabolic syndrome on adverse outcomes.

**Table 3:** Characteristics and outcomes of individuals classified as *No metabolic syndrome*, *At-risk-for metabolic syndrome*, and with *Metabolic syndrome *using individuals with all five criteria measured*

	No metabolic syndrome*	At-risk-for metabolic syndrome*	Metabolic syndrome*	
	**0 criteria**	**1-2 criteria**	**3 or more**	
***Baseline characteristics***				

N = 30,461	9,529	16,674	4,258	
Age, years (SD)	45 (14)	51 (15)	52 (14)	
Women, %	65.8%	59.9%	51.2%	
Race (% White)	76.9%	76.0%	78.1%	
Insurance (% Commercial)	80.2%	66.4%	61.2%	
Income, $US (SD)	$80,859 ($60,623)	$69,846 ($54,333)	$61,086 ($45,290)	

***Three-year outcomes***				p-value †

Inpatient admissions, mean number per patient (SD)	0.10 (0.52)	0.23 (1.07)	0.36 (1.25)	<.0001
Length of stay, mean days (SD) §	4 (3)	6 (3)	6 (3)	<.0001
Outpatient clinic visits, mean number per patient (SD) §	4.3 (2.2)	7.2 (2.5)	11.0 (2.4)	<.0001
Total cost, US dollars (SD) §	$3,804 ($4)	$4,665 ($4)	$5,271 ($4)	<.0001
Incidence of diabetes mellitus	1.1%	3.7%	11.0%	<.0001
Incidence of CHD	2.1%	4.7%	6.5%	<.0001

*patients categorized after excluding patients with diabetes and/or CHD, having all five criteria measured (formal or surrogate); prevalence of each risk factor were 40.8% for obesity, 35.6% for blood pressure, 25.1% for HDL, 18.6% for triglycerides, and 6.7% for glycemia.

† p-values for trend in age-sex-adjusted regression analysis in all three categories of metabolic syndrome status

§log-transformed for analysis and then back-transformed for display

Using our primary approach only 6.6% (5,185 out of 78,293) of the population with at least three risk factors measured was classified as having metabolic syndrome defined as meeting three or more criteria. This low prevalence is due to exclusion of diabetes and CHD before classifying patients with metabolic syndrome, and to missing data. To compare this EHR metabolic syndrome prevalence to national data [3], we performed analyses including patients with diabetes and CHD and with no missing data. Overall, 39,733 patients had all five criteria measured in the 2003-2004 EHR and 9,170 of these (23%) were classified as having *Metabolic Syndrome*, a rate very similar to that reported in the literature for white U.S. adults.

We conducted another set of subsidiary analyses to assess the approach using a more sensitive but less specific cut-off to identify patients with metabolic syndrome (EHR metabolic syndrome diagnosed with two or more criteria present; see Additional file 1: Table S3). Even with this less specific definition of metabolic syndrome, all outcomes were less favorable across metabolic syndrome categories (p-value < 0.0001 for all trends). Using this approach, the PAR of EHR metabolic syndrome was 38.6% for diabetes and 8.0% for CHD risk.

Finally, the sensitivity analysis using all patients in the dataset (shown in Additional file 1: Table S4) confirmed our results in the main analysis: the patients meeting only 1-2 criteria were at increased risk compared to the *No metabolic syndrome *group, even in this larger dataset with more missing values.

## Discussion

Identification of individuals at risk for diabetes and CHD is the first step for primary prevention. Metabolic syndrome has received great fanfare for its putative value to identify at-risk patients for prevention interventions, despite a paucity of data about its actual performance in usual clinical care settings. We have demonstrated here that it is possible to identify patients at risk of developing diabetes and CHD by identifying risk factor clustering using a looser-than-formal metabolic syndrome definition based on combining formal and surrogate criteria available in the EHR of a large primary care network. A looser set of definitions was needed to account for the missing information that is characteristic of usual care data, especially obesity measures and indication of fasting status. Despite missing data, the validation study demonstrated that our approach to define EHR metabolic syndrome was 91% specific to identify patients with formally-diagnosed metabolic syndrome. Even a less specific, more sensitive approach to the definition identified patients at risk for adverse consequences of risk factor clustering.

Using a simple diagnostic algorithm, the patients identified in the EHR as having *Metabolic syndrome *were more than 6 times more likely to develop diabetes, about 42% more likely to develop CHD, and to have higher health resource utilization and health care costs over three years of follow-up compared with individuals identified as not meeting metabolic syndrome diagnosis. The higher health care utilization costs in patients with metabolic syndrome are in accordance with other studies. Curtis *et al*. found that individuals with the metabolic syndrome increased Medicare health care total costs by 20% to 30% [14]. In individuals with all 5 criteria measured, ORs for increased risk of diabetes (OR = 8.67) and CHD (OR = 1.91) are also concordant with published literature: in studies with complete, standardized phenotyping individuals with the metabolic syndrome are about 3-6 times more likely to develop diabetes and to have twice the risk for CHD [1,15,16]. As shown in other reports, our results argue in favor of metabolic syndrome being a stronger predictor of incident diabetes than CHD [16,17]. In addition, we found that patients diagnosed with diabetes or CHD at baseline had about twice the utilization rates and costs compared with patients with EHR metabolic syndrome. These data support the notion that risk factor clustering is identifiable in usual clinical care, is associated with more adverse health outcomes over time, but is less costly than its full-blown diabetes and CHD outcomes. The data argue for the value of risk factor clustering as embodied in metabolic syndrome as a high-risk state amenable to and worthy of detection to prevent transitions from the lower-cost 'risk state' to the higher-cost 'outcome state' of chronic disease management.

Missing data could potentially have biased our findings. Using the data from patients having all five criteria measured and including patients with diabetes and CHD, the prevalence of EHR metabolic syndrome (23%) in our population was very similar to national data: in the NHANES, 20% to 25% of the US population had metabolic syndrome [3]. Once we removed diabetes and CHD, the results from this subsidiary analysis (with all five criteria measured) was very similar to the results using our primary approach (with ≥ three criteria measured). Our approach allowing up to two missing characteristics makes greater use of the available clinical care data with no apparent cost to the validity of the approach. Indeed, our primary algorithm allowed us to classify three-quarters of all patients in the population into one of three metabolic syndrome categories despite the relatively high prevalence of missing data. Since most of the metabolic syndrome criteria (when adapted to include surrogate measures) are typically measured in primary care practice, this means that usual care electronic databases have the potential to be useful clinic population-wide to identify groups of patients at risk for diabetes and CHD. Analysis using individuals with all five criteria measured allowed us to compare our results to other reports and national data of prevalence of metabolic syndrome, but the algorithm using all patients with at least three criteria measured allows identification of a higher number of individuals with metabolic syndrome with high specificity.

Individuals with metabolic syndrome benefit from personalized lifestyle interventions to decrease metabolic abnormalities and prevent diabetes [18]. One of the issues of primary prevention is how to identify patients with "prediabetes" since they are rarely aware of their condition and physicians seldom formally diagnose patients with metabolic syndrome [19]. Our primary, more specific approach (≥ three criteria) allows case-finding for high risk patients for intensive lifestyle interventions to prevent diabetes. Alternatively, the more sensitive but less specific approach (≥ two criteria) identified a larger group of patients and could be useful for larger scale interventions such as targeted screening with information letters or invitations to group education sessions. Our cost data highlight the potential value to health care systems of metabolic syndrome detection for diabetes and CHD prevention.

### Strengths and limitations

Strengths of this study include analysis of a very large number of patients, data from a primary care practice network representing real-world clinical care, and prospective follow-up of outcomes. We included all individuals using health care in the network, with no upper age limit, but only 1% of the patients were 85 years old or above at baseline, so this age range should not affect the main results. We identified diabetes and CHD in the EHR using a validated algorithm, so we are confident that the outcomes represent true incident cases. One limitation was the use of surrogate criteria when the formal criteria were not measured: our validation study showed that EHR metabolic syndrome (using both formal and surrogate criteria) had outstanding specificity (91%) for directly-assessed metabolic syndrome. Missing data were a concern and many of the patients in the *At-risk-for metabolic syndrome *group would probably fall into the category *Metabolic syndrome *if all five criteria would have been available for all patients. This limitation was addressed by an analysis of patients with all five criteria measured that confirmed our primary findings. Also, missing data and misclassification would likely reduce our ability to detect differences between groups, so our primary results probably underestimate actual effects.

## Conclusion

In summary, metabolic syndrome has been extensively studied in highly standardized population samples and national surveys, but very little is known about its prevalence and consequences in real-world primary care practice. Our data shed light on outcomes of free-living patients with metabolic syndrome in usual primary care. EHR data and metabolic syndrome diagnosis both have their limitations, but together they could be a powerful tool to identify patients and populations at risk. Using a simple risk factor clustering algorithm based on metabolic syndrome criteria, EHR can be used to identify individuals at high risk of developing diabetes and CHD and increased health care utilization. We believe that identification of at-risk individuals in this manner should be useful to improve care, target lifestyle interventions for primary prevention of diabetes and CHD, and reduce health care cost and resources utilization associated with risk factor clustering.

## Competing interests

The authors declare that they have no competing interests.

## Authors' contributions

MFH designed and conducted the validation study, participated in the design, collected research data, wrote the manuscript, and contributed to the discussion; RWG participated in the design, contributed to the discussion, and reviewed the manuscript; PS conducted the analysis and reviewed the manuscript; JBM designed the study, contributed to the discussion, and reviewed/edited the manuscript. The authors had full access to the data and take responsibility for its integrity.

## Pre-publication history

The pre-publication history for this paper can be accessed here:



## Supplementary Material

Additional file 1 **Supplementary tables**. tables S1, S2, S3, and S4 are included in additional file 1. **Table S1**. Results of the validation study in 154 patients recruited in the Internal Medicine Associates (IMA) practice at Massachusetts General Hospital. **Table S2**. Numbers and percentage of individuals without diabetes or CHD meeting formal and surrogate metabolic syndrome criteria in electronic health record data (in all patients; in patients with at least 3 criteria measured; and in patients with all 5 criteria measured). **Table S3**. Characteristics and outcomes of individuals using metabolic syndrome defined as meeting two or more criteria (a more sensitive approach than meeting three or more criteria) using individuals with at least three criteria measured in the electronic health record. **Table S4**. Three-year outcomes (2005-2007) for individuals with *No metabolic syndrome*, *At-risk-for metabolic syndrome*, with *Metabolic syndrome*, and with CHD/DM according to electronic health record data in all patients. Click here for file
