# Supplementary material for: Identifying primary care patients at risk for future diabetes and cardiovascular disease using electronic health records
Source: BMC Health Serv Res. 2009 Sep 22;9:170. doi: 10.1186/1472-6963-9-170 (PMC2753330; doi:10.1186/1472-6963-9-170)
Supplement: Additional file 1 — Supplementary tables. tables S1, S2, S3, and S4 are included in additional file 1. Table S1. Results of the validation study in 154 patients recruited in the Internal Medicine Associates (IMA) practice at Massachusetts General Hospital. Table S2. Numbers and percentage of individuals without diabetes or CHD meeting formal and surrogate metabolic syndrome criteria in electronic health record data (in all patients; in patients with at least 3 criteria measured; and in patients with all 5 criteria measured). Table S3. Characteristics and outcomes of individuals using metabolic syndrome defined as meeting two or more criteria (a more sensitive approach than meeting three or more criteria) using individuals with at least three criteria measured in the electronic health record. Table S4. Three-year outcomes (2005-2007) for individuals with No metabolic syndrome, At-risk-for metabolic syndrome, with Metabolic syndrome, and with CHD/DM according to electronic health record data in all patients. [file 1472-6963-9-170-S1.DOC]

| **Table S1.** Results of the validation study in 154 patients recruited in the Internal Medicine Associates (IMA) practice at Massachusetts General Hospital (MGH)* | | | |
| --- | --- | --- | --- |
|  | sensitivity | specificity | c-statistic† |
| Central obesity | 88.3% | 86.8% | 0.876 |
| Elevated blood pressure | 73.4% | 86.7% | 0.800 |
| Elevated glucose | 49.1% | 86.4% | 0.678 |
| Elevated triglycerides | 61.1% | 90.9% | 0.760 |
| Low HDL cholesterol | 77.1% | 93.9% | 0.855 |
| Metabolic Syndrome  (defined as ≥3 criteria) | 73% | 91% | 0.818 |

*Performance of the approach using formal and surrogate criteria in the electronic health records (EHR) of patients with at least three criteria measured compared to direct phenotyping of all criteria in a standardized fashion.

† Area Under the Receiver Operating Characteristic (ROC) Curve

**Table S2. Numbers and percentage of individuals without diabetes or CHD meeting formal and surrogate metabolic s**yndrome criteria in electronic health record data

|  | In all patients* | | >3 measured criteria† | | All 5 criteria measured ‡ | |
| --- | --- | --- | --- | --- | --- | --- |
|
|  | n | % | n | % | n | % |
| Total | 105,395 |  | 78,293 |  | 30,461 |  |
| **Obesity** |  |  |  |  |  |  |
| Formal measured |  |  |  |  |  |  |
| meeting formal criterion | 0 | 0.0% | 0 | 0.0% | 0 | 0.0% |
| not meeting formal | 0 | 0.0% | 0 | 0.0% | 0 | 0.0% |
| Surrogate measured |  |  |  |  |  |  |
| meeting surrogate criterion | 21197 | 20.1% | 18242 | 23.3% | 12430 | 40.8% |
| not meeting surrogate | 40867 | 38.8% | 31801 | 40.6% | 18031 | 59.2% |
| Missing | 43331 | 41.1% | 28250 | 36.1% | n/a | n/a |
| Total meeting criteria | 21197 | 20.1% | 18242 | 23.3% | 12430 | 40.8% |
| **Blood pressure** |  |  |  |  |  |  |
| Formal measured |  |  |  |  |  |  |
| meeting formal criterion | 20005 | 19.0% | 17714 | 22.6% | 10828 | 35.6% |
| not meeting formal | 85390 | 81.0% | 60579 | 77.4% | 19633 | 64.5% |
| Surrogate measured |  |  |  |  |  |  |
| meeting surrogate criterion | n/a | n/a | n/a | n/a | n/a | n/a |
| not meeting surrogate | n/a | n/a | n/a | n/a | n/a | n/a |
| Missing | n/a | n/a | n/a | n/a | n/a | n/a |
| Total meeting criteria | 20005 | 19.0% | 17714 | 22.6% | 10828 | 35.6% |
| **HDL** |  |  |  |  |  |  |
| Formal measured |  |  |  |  |  |  |
| meeting formal criterion | 15622 | 14.8% | 15562 | 19.9% | 7632 | 25.1% |
| not meeting formal | 53369 | 50.6% | 53129 | 67.9% | 22775 | 74.8% |
| Surrogate measured |  |  |  |  |  |  |
| meeting surrogate criterion | 309 | 0.3% | 298 | 0.4% | 10 | 0.03% |
| not meeting surrogate | 1105 | 1.0% | 1057 | 1.4% | 44 | 0.1% |
| Missing | 34990 | 33.2% | 8247 | 10.5% | n/a | n/a |
| Total meeting criteria | 15931 | 15.1% | 15860 | 20.3% | 7642 | 25.1% |
| **Triglycerides** |  |  |  |  |  |  |
| Formal measured |  |  |  |  |  |  |
| meeting formal criterion | 5595 | 5.3% | 5595 | 7.2% | 3005 | 9.9% |
| not meeting formal | 16520 | 15.7% | 16519 | 21.1% | 7870 | 25.8% |
| Surrogate measured |  |  |  |  |  |  |
| meeting surrogate criterion | 5106 | 4.8% | 5106 | 6.5% | 2659 | 8.7% |
| not meeting surrogate | 34274 | 32.5% | 34274 | 43.8% | 16927 | 55.6% |
| Missing | 43900 | 41.7% | 16799 | 21.5% | n/a | n/a |
| Total meeting criteria | 10701 | 10.2% | 10701 | 13.7% | 5664 | 18.6% |
| **Glucose** |  |  |  |  |  |  |
| Formal measured |  |  |  |  |  |  |
| meeting formal criterion | 3635 | 3.4% | 3607 | 4.6% | 1794 | 5.9% |
| not meeting formal | 14461 | 13.7% | 14207 | 18.2% | 7339 | 24.1% |
| Surrogate measured |  |  |  |  |  |  |
| meeting surrogate criterion | 803 | 0.8% | 695 | 0.9% | 262 | 0.9% |
| not meeting surrogate | 55498 | 52.7% | 51001 | 65.1% | 21066 | 69.2% |
| Missing | 30998 | 29.4% | 8783 | 11.2% | n/a | n/a |
| Total meeting criteria | 4438 | 4.2% | 4302 | 5.5%  %% | 2056 | 6.7% |

*after exclusion of diabetes and/or cardiovascular disease (CHD); percentages are expressed using the total number of patients without diabetes/CHD (n=105,395)

† after exclusion of diabetes/CHD, and exclusion of patients with less than three measured criteria (formal or surrogate); percentage are expressed using the total number with three or more measured criteria (n=78,293)

‡ after exclusion of diabetes/CHD, percentages are expressed using the total number with all five criteria measured (n=30,461)

n/a: non applicable, all patients have been classified in previous categories (all patients had blood pressure measurements, by definition, no missing data in the patients with all five criteria measured)

**Table S**3. Characteristics and outcomes of individuals using metabolic syndrome defined as meeting two or more criteria (a more sensitive approach than meeting three or more criteria) using individuals with at least three criteria measured in the electronic health record*

|  | No metabolic syndrome | At-risk-for metabolic syndrome | Metabolic syndrome |  |
| --- | --- | --- | --- | --- |
|
|  | 0 criteria | 1 criteria | 2 or more |  |
| ***Baseline characteristics*** | | | |  |
| n=78 293 | 36,841 | 23,927 | 17,525 |  |
| Age, years (SD) | 45 (15) | 49 (16) | 51 (15) |  |
| Women, % | 62.8% | 58.5% | 53.2% |  |
| Race (% White) | 78.8% | 76.7% | 76.8% |  |
| Insurance (% Commercial) | 77.4% | 67.0% | 63.7% |  |
| Income, $US (SD) | $81,402 ($59,796) | $72,575 ($54,845) | $65,295 ($49,160) |  |
| ***Three-year outcomes*** | | | | p-value † |
| Inpatient Admissions, mean number per patient (SD) | 0.12 (0.61) | 0.22 (1.06) | 0.29 (1.12) | <.0001 |
| Length of stay, mean days (SD) § | 4 (3) | 5 (3) | 6 (3) | <.0001 |
| Outpatient Clinic Visits, mean number per patient (SD) § | 4.3 (2.3) | 6.0 (2.5) | 8.5 (2.5) | <.0001 |
| Total Cost, US dollars (SD) § | $3,054 ($4) | $3,937 ($4) | $4,691 ($4) | <.0001 |
| Incidence of Diabetes | 1.4% | 3.0% | 7.3% | <.0001 |
| Incidence of CHD | 3.2% | 4.9% | 6.1% | <.0001 |

* Individuals categorized after exclusion of diabetes and/or CHD, including only patients with at least three criteria (formal or surrogate) measured (n=78,293) using electronic health record data from 2003-2004

† p-values for trend in age-sex-adjusted regression analysis in all 3 categories of metabolic syndrome status

§ log-transformed for analysis and then back-transformed for display

| **Table S4. Three-year outcomes (2005-2007) for individuals with *No metabolic syndrome*, *At-risk-for metabolic syndrome*, with *Metabolic syndrome*, and with CHD/DM according to electronic health record data in all patients** | | | | | | |
| --- | --- | --- | --- | --- | --- | --- |
|
|  | No metabolic syndrome* | At-risk-for metabolic syndrome* | Metabolic syndrome* | Any CHD/DM | p-value † | p-value ‡ |
|  | 0 criteria | 1-2 criteria | >3 criteria |  |  |  |
| N=122,715 | 59 120 | 41 090 | 5 185 | 17 320 |  |  |
|  |  |  |  |  |  |  |
| ***Three-year outcomes*** |  |  |  |  |  |  |
| Inpatient admissions, mean number per patient (SD) | 0.10 (0.56) | 0.22 (1.04) | 0.35 (1.20) | 1.5 (3.6) | <.0001 | <.0001 |
| Length of stay, mean days (SD) § | 4 (3) | 6 (3) | 6 (3) | 12 (3) | <.0001 | <.0001 |
| Outpatient clinic visits, mean number per patient § | 3.6 (2.4) | 6.1 (2.6) | 10.4 (2.5) | 20.5 (2.5) | <.0001 | <.0001 |
| Total cost, US dollars (SD) § | $2,714 ($4) | $3,942 ($4) | $5,057 ($4) | $9,177 ($4) | <.0001 | <.0001 |
| Incidence of diabetes mellitus | 1.3% | 3.7% | 11.0% | N/A | <.0001 | N/A |
| Incidence of CHD | 2.5% | 4.9% | 6.4% | N/A | <.0001 | N/A |

* Individuals categorized after exclusion of diabetes and/or CHD

† p-values for trend in age-sex-adjusted regression analysis in all 3 categories of metabolic syndrome status

‡ p-values for trend in age-sex-adjusted regression analysis in all 4 categories

§ log-transformed for analysis and then back-transformed for display
